# Recent advance on nanoparticles or nanomaterials with anti-multidrug resistant bacteria and anti-bacterial biofilm properties: A systematic review

**DOI:** 10.1016/j.heliyon.2023.e22105

**Published:** 2023-11-10

**Authors:** Farhad Moradi, Arshin Ghaedi, Zahra Fooladfar, Aida Bazrgar

**Affiliations:** a Department of Bacteriology & Virology, School of Medicine, Shiraz University of Medical Sciences, Shiraz, Iran; b Student Research Committee, School of Medicine, Shiraz University of Medical Sciences, Shiraz, Iran

**Keywords:** Nanoparticles, Nanomaterial, Multidrug Resistant, Bacterial biofilm, Systematic review

## Abstract

**Objective:**

With the wide spread of Multidrug-resistant bacteria (MDR) due to the transfer and acquisition of antibiotic resistance genes and the formation of microbial biofilm, various researchers around the world are looking for a solution to overcome these resistances. One potential strategy and the best candidate to overcome these infections is using an effective nanomaterial with antibacterial properties against them.

**Methods:**

*and analysis*: In this study, we overview nanomaterials with anti-MDR bacteria and anti-biofilm properties. Hence, we systematically explored biomedical databases (Web of Sciences, Google Scholar, PubMed, and Scopus) to categorize related studies about nanomaterial with anti-MDR bacteria and anti-biofilm activities from 2007 to December 2022.

**Results:**

In total, forty-one studies were investigated to find antibacterial and anti-biofilm information about the nanomaterial during 2007–2022. According to the collected documents, nineteen types of nanomaterial showed putative antibacterial effects such as Cu, Ag, Au, Au/Pt, TiO2, Al2O3, ZnO, Se, CuO, Cu/Ni, Cu/Zn, Fe3O4, Au/Fe3O4, Au/Ag, Au/Pt, Graphene O, and CuS. In addition, seven types of them considered as anti-biofilm agents such as Ag, ZnO, Au/Ag, Graphene O, Cu, Fe3O4, and Au/Ag.

**Conclusion:**

According to the studies, each of nanomaterial has been designed with different methods and their effects against standard strains, clinical strains, MDR strains, and bacterial biofilms have been investigated in-vitro and in-vivo conditions. In addition, nanomaterials have different destructive mechanism on bacterial structures. Various nanoparticles (NP) introduced as the best candidate to designing new drug and medical equipment preventing infectious disease outbreaks by overcome antibiotic resistance and bacterial biofilm.

## 1 Introduction

Bacterial infectious illnesses account for around one-third of worldwide mortality [1,2]. Preventing infectious disease outbreaks is a constant issue due to the relatively low infection dosage, about 100 organisms, and the biological variety of dangerous pathogens [[1], [2], [3]]. Antibiotics have been employed to treat harmful bacterial infections since the 1940s. One of the most effective strategies for lowering human morbidity and death is using antibiotics in the medical profession [3,4]. Since the discovery of penicillin in the 1940s, antibiotics have saved countless human lives while also permitting current medical operations. Consequently, antibiotics have become economic powerhouses in the world, with over 42 billion US dollars in antibiotic sales in 2009, accounting for almost 15–30 % of total medication spending across all therapeutic classes of pharmaceuticals [[3], [4], [5]]. However, due to the widespread use of antibiotics (100,000 tons per year globally), human infections have developed resistance to many medicines like antibiotics [[4], [5], [6], [7]]. The accumulation of transposons or plasmids harboring resistant genes in different strains causes the appearance of MDR bacteria [5]. Nowadays, the emergence of some bacteria with high resistance to antibiotics, such as MDR *Pseudomonas aeruginosa*, vancomycin-resistant *Enterococcus* (VRE), carbapenem-resistant *Acinetobacter* species, Methicillin-resistant *Staphylococcus aureus* (MRSA), carbapenem-resistant Enterobacterales, extended-spectrum cephalosporin resistance in Enterobacterales is suggestive of extended spectrum beta-lactamase (ESBL) production has become a serious threat to humans. Hence, every year, various researchers around the world conduct extensive research to breakdown these types of resistance. MDR bacteria have shown resistance to five commonly used antibiotics, including chloramphenicol, ampicillin, streptomycin, tetracycline, and sulfonamides [3,5,7]. Moreover, these MDR bacteria are responsible for 60 % of nosocomial infections. It will not be easy to create entirely new antibiotics, given the state of the global economy today [8]. Since antibiotics, unlike all other types of medications, have a finite shelf life, the day will soon come when we can no longer safely treat bacterial illnesses with the present antibiotics [7,8]. The World Health Organization (WHO) estimates that there may be another 1-2 decades for humans to utilize the present medicines until infectious illnesses caused by MDR bacteria are no longer treatable with current antibiotics [9]. This makes it abundantly evident that the need for novel, non-traditional therapeutic methods for treating pathogenic bacterial diseases is of the greatest priority [9]. One potential approach, yet in primary stages, is the treatment of MDR bacteria based on nanomaterials [[10], [11], [12], [13], [14], [15], [16], [17]]. Another problem about bacteria we are facing nowadays is bacterial biofilms. Complex communities of bacteria that are encased in a matrix and exhibit higher antibiotic resistance, as well as immune system evasion, are known as biofilms [18]. They are capable of causing infections that are resistant to conventional antibiotic treatment. According to the National Institutes of Health (NIH), many microbial infections are associated with biofilm formation [19]. Hence, with the wide spread of antibiotic resistant strains due to the transfer and acquisition of antibiotic resistance genes or the formation of microbial biofilm, various researchers around the world are looking for a solution to overcome these resistances. Today, various methods and compounds suggested replacing antibiotics, such as quorum quenching, phage therapy, photodynamic therapy, host direct therapy, etc. Natural compounds include herbal medicine or traditional plants. Many studies documented the use of medicinal plants alone or in combination with antibiotics can overcome antibiotic resistance [17]. For example, quorum quenching or interference in the signaling systems of drug-resistant bacteria using compounds of natural origin or synthetic compounds. In this technique, instead of destroying the microbial structure, similar to the action of antibiotics, the signal exchange among the microbial population is inhibited, and the bacteria are unable to express genes associated with invasion or biofilm formation [18]. Also, phage therapy, is one of the new achievements to control microbial biofilm and overcome antibiotic resistance, especially for MRSA [19]. Other technique include the design of nucleic acid aptamers. In fact, aptamers can overcome microbial resistance by inhibiting the translation of mRNA associated with proteins or enzymes effective in antibiotic resistance [20]. On the other hand, other researchers introduced photodynamic therapy technique as a promising solution in controlling resistant microbial strains and destroying the biofilm structure. In this technique, a putative light source by stimulating photosensitizer can produce destructive free radicals. These free radicals can react with different types of macromolecules and biological structures, especially in biofilm, and destroy them [21]. Although the mentioned methods have received a lot of attention and are in-vitro, in-vivo, and clinical trials stages, but due to the need for special materials, equipment, laboratory experiences, they have not yet reached the practical stage in treatment and clinical practice. In addition, today the science of nanotechnology has created a great revolution in the field of medicine and treatment by providing and examining different nanostructures with antibacterial properties. For instance, lipid or polymer nanoparticles (NPs) are viable methods for drug administration to overcome biofilm resistance. These particles may enhance antibiotic transport to bacterial cells, improving the effectiveness of the therapy [22]. With the efficiency of existing chemotherapeutics deteriorating owing to rising drug resistance, there is an urgent need to develop alternative medicines. One of the most recent initiatives to combat bacterial and biofilm resistance has been to investigate antimicrobial nanomaterial to which microbial pathogens may be unable to acquire resistance, as well as innovative nano-sized platforms for effective antibiotic delivery [21]. Nanomaterial have shown promise in this regard due to their particular chemical and physical properties [[22], [23], [24]]. Their huge surface area to volume ratio allows for close interactions with microbial membranes and surface functionalization, which aids in developing more potently antibacterial agents [25]. Recent investigations, for example, have revealed that certain metal nanoconstructs have antibacterial properties that may be used to manage infectious disorders [[26], [27], [28]]. Over the past decade, there has been a tremendous worldwide emphasis on conventional and biogenic metallic NPs as novel methods for tackling antimicrobial resistance [23,29]. Chemotherapeutic medications combined with metallic NPs may have a cumulative impact. Furthermore, the antibacterial agent may be employed at a considerably lower dosage than when used alone, overcoming resistance and reducing other unpleasant side effects to some degree [23,29]. A paradigm change has also occurred in managing MDR and biofilm bacteria using antibiotic-loaded polymeric NPs and polymeric nanocomposites [25]. Given the significance of this topic, we chose to summarize our extensive searches from online databases from June 2007 to December 2022, which we discovered to be successful in clarifying the antibacterial activity of nanomaterial against MDR bacteria and biofilm. In the present review, we have tried to review the nanomaterials whose antibacterial and anti-biofilm effects have been shown by different researchers.

## 2 Material and methods

### 2.1 Search strategy

**Fig. 1:**
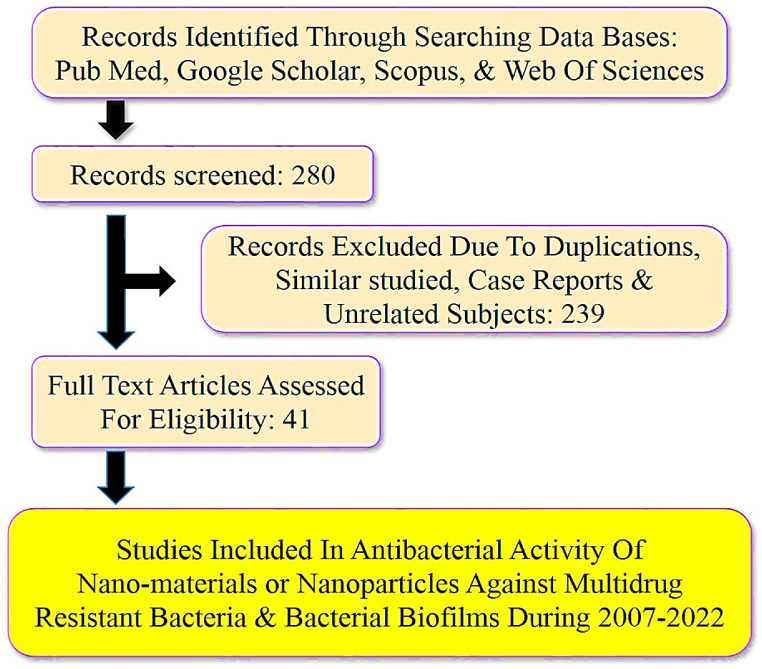
Flow chart of search process and study selection.

We systematically searched biomedical databases (Web of Sciences, PubMed, Google Scholar, and Scopus) to identify related studies that were published until December 2022. The search was performed using different combinations of the following keywords:” Nanomaterial AND Antibacterial activity OR Antimicrobial activity”, “Nanoparticle AND Antibacterial activity OR Antimicrobial activity”, “Nanomaterial OR Nanoparticle AND Multidrug-resistant (MDR) bacteria”, “Nanomaterial OR Nanoparticle AND Gram-positive Multidrug-resistant (MDR) bacteria”, “Nanomaterial OR Nanoparticle AND Gram-negative Multidrug-resistant (MDR) bacteria”, “Nanomaterial OR Nanoparticle AND Resistant bacteria”, “Nanomaterial OR Nanoparticle AND Anti-Biofilm activity”, “Nanomaterial OR Nanoparticle AND Bacterial Biofilm”. Moreover, additional information were collected from the references of the included studies and the quality assessment was performed according to the Joanna Briggs Institute (JBI) checklist. Finally, out of 280 recognized articles, 41 papers were identified that were published between 2007 and 2022 (Fig. 1.).

### 2.2 Inclusion and exclusion criteria

To clarify the nanomaterials or NPs with antibacterial properties, we reviewed the literature that was published based on the antibacterial activity of some nanomaterials or NPs against gram-positive and gram-negative resistant bacteria, MDR bacteria, and bacterial biofilms as inclusion criteria. Information about nanomaterials or NPs with antibacterial activities in different studies was characterized based on the type of NPs, type of bacterial strains or biofilms, antibacterial mechanisms or inhibition pathways, and effective application of each nanoparticle according to the suggestion of literature authors. Furthermore, all of the data and publications were evaluated based on their topic, time, and resources to generate a review that may be beneficial for future research or assist physician's better handling MDR bacteria and biofilm infection (Table 1). Also, studies that did not explore the antibacterial or anti-biofilm activity of nanomaterial's, any type of research related to nanomaterial with non-antibacterial properties (for example, with antifungal, antiviral, and anti-parasitic effects), and similar studies related to investigating the antibacterial effects of a specific nanoparticle. In addition, studies that were conducted to investigate the effects of NPs other than antibacterial and anti-biofilm, case reports studies and duplicate documents were considered as exclusion criteria.

**Table 1:** Nanoparticles or Nanomaterials with anti-Multidrug Resistant Bacteria and anti-biofilm Properties during 2007–2022.

Type of NPs	First author/publication yea	Targeted bacteria/Antibiotic resistance type/Biofilm	Antibacterial effects or pathway	Author suggestions	References
Ag NPs	Xiu Z-m/2012	Gram-negative bacteria, *E. coli*	The toxicity of various Ag NPs accurately follows the dose-response pattern of *E. coli* exposed to Ag (+).	This work suggests that AgNP morphological properties known to affect antimicrobial activity are indirect effectors that primarily influence Ag + release.	[30]
	Esmaeillou M/2017	MDR organisms such as VRE and MRSE	Antibacterial activity by The MIC was 0.1 μg/ml for VRE, MIC ≤0.02 μg/ml for MRSE, and 0.05 μg/ml for *S. aureus.*	Some antimicrobial agents like vancomycin can be conjugated with Ag NPs and increased antimicrobial activity against MDR microorganisms.	[31]
	Mohanta YK/2020	antibacterial and anti-biofilm activity against *P. aeruginosa* MTCC 741, *E. coli* MTCC 739, and *S. aureus* MTCC 96	Plant synthesized AgNPs at different concentrations (10–100 μg/ml) have an anti-biofilm activity and toxic activity against *S. aureus*, *P. aeruginosa,* and *E. coli*.	Plant (*S. anacardium, Glochidion lanceolarium, and Bridelia retusa*)-derived Ag NPs have potential to inhibit biofilm formation and effectively treating a variety of infectious diseases.	[32]
	Lara HH/2010	MDR *P. aeruginosa*, ampicillin-resistant *E. coli* O157:H7, erythromycin-resistant *S. pyogenes*	Bactericidal action based on inhibition of cell wall, protein, and nucleic acid synthesis.	Best candidate for use in pharmaceutical products and medical devices.	[33]
	Thapa R/2017	Extended spectrum beta-lactamase (ESBL) positive *Escherichia coli*, *S. aureus* (MRSA), Teicoplanin resistant *S. Pneumoniae.*	Showed antibacterial activity through disc diﬀusion method	The nano-silver treated nylon thread can be useful especially for the stitching the surgery cuts to avoids the secondary infection.	[34]
	Percival S/2007	A wide variety of antibiotic-resistant bacteria and *C. albicans* known to be associated with wound colonization and infection.	Silver-containing Hydro fiber (SCH) dressing was most effective against *P. aeruginosa,* *C. albicans* and *S. aureus* (ZOI range: 2·6–6 mm). The nano crystalline silver-containing (NCS) dressing was most effective against *K. pneumoniae*, *E. faecalis* and *E. coli* (ZOI range: 1–2·8 mm).	The SCH dressing demonstrated greater biofilm-inhibiting activity than the NCS.	[35]
Au NPs	Zheng K/2017	Gram-positive and Gram-negative bacteria	Ultra small Au nano clusters (NCs) could induce a metabolic imbalance and increase of intracellular reactive oxygen that kills bacteria consequently.	It is possible to confer antimicrobial activity to Au NPs through precise control of their size down to NC dimension (typically less than 2 nm).	[36]
	Sathiyaraj, S/2021	B. subtilis, *K. pneumoniae*	The Au NPs showed antibacterial activity against *K. pneumoniae* & B. subtilis.	Using panchagavya to synthesize gold, NPs have strong toxicity against gram-negative bacteria in compare to gram-positive bacteria.	[37]
TiO_2_ NPs	Thakur, B/2019	*S. typhi, E. coli,* *K. pneumoniae.*	The lowest MIC value was observed against *S. typhi* and *E. coli* whereas lowest MBC value was observed against *K. pneumoniae.*	Using *Azadirachta indica* leaf extract to synthesis of titanium dioxide NPs exhibited broad-spectrum antimicrobial activity as compared with TiO2 compound.	[38]
	Simon-Deckers A/2009	*E*. *coli* MG1655	Reactive oxygen generation and impairment of cell membrane integrity	Ti O_2_ toxicity depends on their chemical composition, size, surface charge, and shape and bacterial strain but not on their crystalline phase or purity	[39]
	Li Y/2012	Examined the viability of *E. coli* cells in aqueous suspensions of NPs under UV irradiation	Light-enhanced antibacterial activity of Ti O_2_ by oxidative stress that induced by ROS	Ti O_2_ yielded more ROS than their bulk counterparts likely due to larger surface areas.	[40]
	Roy AS/2010	clinical isolate of MRSA	Antibacterial activity of different antibiotics such as beta lactams and cephalosporins can be increased by sub-MIC of TiO2 NPs	TiO2 NP showed a synergic effect on the antibacterial activity of nalidixic acid against MRSA.	[41]
Al_2_O_3_ NPs	Ansari MA/2013	MRSA and methicillin-resistant coagulase negative staphylococci	Disruption of cell membrane and cell wall, Penetrated and interact with the cellular macromolecules.	In depth studies regarding the interaction of the NPs with cells, tissues, and organs as well as the optimum dose required to produce therapeutic effects need to be ascertained	[42]
	Ansari MA/2014	MDR clinical isolates of *E. coli*	Induced perforation and destruction of cell wall.	Further study need to assessment of the biologically relevant dose, toxic impact on eukaryotic system.	[43]
ZnO NPs	Aldeen T. S/2022	*S. aureus*, S. pneumonia, *E. coli*, *S. typhi*	Significant bactericidal effect towards gram-positive and gram-negative pathogenic bacteria.	The aqueous extract of Phoenix roebelenii palm leaves has been used to synthesize ZnO NPs via a green chemistry approach with exhibit antibacterial activity.	[44]
	Raghupathi KR/2011	*S. aureus* & *E. coli*	ROS generation	ZnO *N*P-containing formulations may be utilized as antibacterial agents in ointments, lotions, mouthwashes.	[45]
	Cha *S*–H/2015	MDR *E. coli*, MRSA	ROS generation, Enzyme inhibition (β-galactosidase), & disruption of bacterial cell wall	small zinc oxide NPs pyramids, plates, and spheres inhibit activity of bacterial β-galactosidase in a biomimetic fashion	[46]
	Reddy LS/2014	*K. pneumoniae* ATCC 70068	Disruption of bacterial cell wall and blocking bacterial invasion	Suggest the potential antibacterial against *K. pneumoniae* infections.	[47]
	B Aswathanarayan J/2017	MRSA, *P. aeruginosa* PA01, Biofilm inhibitory	The ZnO NPs had an MIC in the range of 3.125 μg/ml and 6.25 μg/ml against the tested pathogens.	ZnO NPs had significant antimicrobial activity against MDR pathogens with less toxicity to mammalian cells in comparison to gold and iron nanoparticle.	[48]
Au–Ag NPs	Ding X/2017	*S*. *aureu*s, Bacterial biofilm	Antibacterial activity, negligible toxicity to human dermal fibroblasts, Removed bacterial biofilm under NIR irradiation,	Light-enhanced antibacterial Au and Au@Ag NPs can potentially be used as imaging and antibacterial agents.	[49]
Cu NPs	Lewis Oscar F/2015	*P. aeruginosa*	Anti-biofilm activity	Cu NPs could be utilized as coating agents on surgical devices and medical implants to manage biofilm-associated infections.	[50]
	Chen S/2017	Gram-positive & negative bacteria	ROS generation with low toxicity to NIH 3T3 cells	Temperature increase caused by the photo thermal conversion of Cu NPs in the hydrogel induced the antibacterial behavior.	[51]
Copper sulfide nanoclusters	Dai X/2017	levofloxacin-resistant *S. aureus, E. coli,* *P. aeruginosa*, & *B. amyloliquefaciens*	Inhibit bacterial species by heat and ROS generated under NIR laser irradiation	CuS NPs and CuSNPs plus NIR showed obvious cytotoxicity to cells when the concentration was up to 88 μg/ml, suggesting PATA3-C4 plays an important role in improving the biocompatibility of CuS NPs.	[52]
Cu O-NPs	Chakraborty R/2015	MDR *E. coli & S. aureus*	ROS generation, destroy bacterial DNA	‘particle-specific’ effect, not ‘ion-specific’ one, was responsible for the NP action.	[53]
Cu/Ni bimetallic NPs	Argueta-Figueroa L/2014	*S. aureus, E. coli*, *S. mutans*	Cu NPs show a bactericidal effect while Ni NPs and bimetallic Cu–Ni NPs exhibit only bacteriostatic effects.	These NPs have promising properties for applications in dentistry.	[54]
Cu/Zn in carbon nanofibers	Ashfaq M/2015	*E. coli, S. aureus,* MRSA	Suppressed the growth of bacteria throughout different release profiles.	Possibly in wound healing and wound dressing.	[55]
Se NPs	Zhao Z/2017	MRSA	SiO2-Cy-Van nano probes exhibit photo thermal antimicrobial effects by disruptions of the bacterial cell wall.	These nano-probes can be useful for tracking bacterial load in infected tissues.	[56]
	Huang N/2017	*S. aureus* ATCC 6538, *E. coli* ATCC 8739	ROS generation via oxidative damage to macromolecules and destroy bacterial cell wall	Ruthenium complexes/polypeptide self-assembled (Se@PEPRu) NPs can be used to distinguish between bacterial infections and tumor-induced tissue infection with high specificity.	[57]
Fe3O4NPs	Chaurasia AK/2016	MDR *S. aureus* USA300, UPEC CFT073, and their Biofilms	Induce bacterial membrane dysfunction	Maximum killing efficiency was obtained when bacteria were treated with MCSNPs in combination with radiofrequency exposure and physical treatment.	[58]
	Behera S/2012	*S. aureus* MTCC 1144*,* *B. licheniformis* MTCC 7425, *B.brevis* MTCC 7404, *V.cholerae* MTCC 3904, *P. aeruginosa* MTCC 1034, *S. epidermidis* MTCC 3615*, B. subtilis* MTCC 7164, *E. coli* MTCC 1089.	ROS generation that cause damage to biological macromolecule by oxidative stress	Fe O NPs shows better bactericidal activity in Gram-positive bacteria as compared to Gram-negative bacteria and can be explored for its topical application in pharmaceutical and biomedical industries.	[59]
	El Zowalaty ME/2015	*S. aureus* ATCC43300), *P. aeruginosa (*ATCC27853)*,* aeruginosa (ATCC *E.coli* (ATCC25922), *C. albicans* (ATCC 20408), *M. tuberculosis* ATCC25618)	Magnetic NPs (Fe (3+)) coated by chitosan and loaded by Streptomycin showed The antibacterial effect for *M. tuberculosis* and more active *M. tuberculosis* was 732 μg/ml against Gram-negative bacteria as compared to Gram-positive bacteria.	New type of magnetic nanoparticle with microorganism theranostic properties as a potential tool to both diagnose and treat diverse microbial and tuberculosis infections.	[60]
Fe₃O₄@AuNPs	Niemirowicz K/2014	MDR *P. aeruginosa*	Damage to the bacterial cell wall, Interacts with bacterial proteins, and generation of ROS.	Fe3 O4 @Au significantly reduce the growth of *P. aeruginosa* and have a compatibility with human healthy cells.	[61]
Au/Ag bimetallic NPs	Zhou Z/2018	VRE	Designed nano system (Au@AgNP@SiO_2_@Nc-Van) revealed Van-enhanced specific binding affinity toward VRE and photo inactivate VRE.	This novel nanosystem has huge potential for applications in theranostics with regard to VRE management.	[62]
	Ramasamy M/2016	*E. coli, S. aureus, E. faecalis, P. aeruginosa*/Biofilm formation	Inactivate the proteins and enzymes for ATP production	Promising nanoantibiotic by using *γ*-proteobacterium, *Shewanella oneidensis* MR-1can be effective to overcoming the bacterial resistance in the established bacterial biofilms.	[63]
Au/Pt bimetallic NP	Zhao Y/2014	MDR *E. coli*	Damage bacterial inner membrane	Au Pt NPs showed selective toxicity to bacteria but not to mammalian cells	[64]
Graphene Oxide NPs	Menazea A.A/2020	*E. coli*, *S. aureus*	GO/Ag NPs and GO/CuO NPs have demonstrated outstanding antibacterial activity.	Graphene oxide have been decorated by silver and copper oxide NPs are suitable for biomedical application.	[65]
	Pan W–Y/2016	MRSA	Reduced graphene oxide (rGO)-iron oxide NPs (rGO-IONP under near-infrared laser inactivated MRSA	This nanocomposite system can effectively inactive MDR bacteria in subcutaneous infections	[66]
	Kulshrestha S/2017	Biofilm formation in both E. *cloacae* and S. *mutans*	Anti-biofilm activity at the sub-inhibitory concentrations.	Biofilm inhibitory concentrations of GO-Ag NP were also found to be non-toxic against HEK-293 cell line.	[67]
	Govindaraju S/2016	*E. coli*, *E. faecalis*	UV-irradiated Glucosamine-gold nanoaprticle-graphene oxide (GlcN-AuNPs-GO) has better antibacterial activity than normal GlcN-AuNP-GO and kanamycin.	UV irradiated GlcN-AuNP-GO showed the highest ROS value of all nanomaterials.	[68]
Ni-NPs	Ahghari M.R./2020	*S. aureus*, *E. coli*.	Penetrated inside the bacteria, and destroyed DNA.	The nickel magnetic mirror NPs can be applied in domestic usages such as dentistry, surgery, and space telescopes.	[69]
Si O2	Alavi M/2022	MDR bacteria.	Modification or functionalization of silica NPs by conventional antibiotics, metal/metal oxide NPs and biodegradable polymers showed increase the bactericidal activity.	Green synthesize silica NPs loading antibiotic would be a very effective solution for the silent pandemic of antibiotic resistance, case of chronic, bacterial infections specifically diabetic foot ulcer, pneumonia, and *pseudomonas* infections.	[70]

## 3 Results

**Fig. 2:**
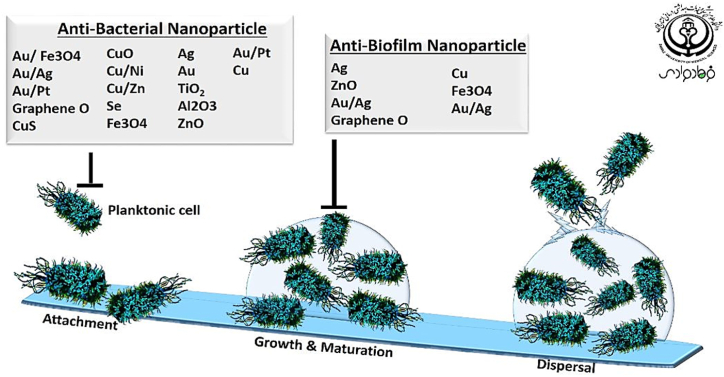
According to the studies, nineteen types of nanomaterial showed antibacterial effects, and seven types of them are considered anti-biofilm agents.

In total, forty-one studies were investigated to find antibacterial and anti-biofilm information on the nanomaterial during 2007–2022. These data were categorized and accessible in Table 1. According to the collected documents, nineteen types of nanomaterial showed putative antibacterial effects, and seven types of them are considered anti-biofilm agents (Fig. 2.). Different studies examined the antibacterial effects of the nanoparticle using three types of bacteria. One of them is various standard strains of gram-negative and gram-positive bacteria without antibiotic resistance such as *Pseudomonas aeruginosa* ATCC27853, *Staphylococcus aureus* ATCC 6538, *Klebsiella pneumoniae* ATCC 70068, *Escherichia coli* ATCC 8739, and *Mycobacterium tuberculosis* ATCC25618. The second mode is the use of bacterial strains isolated from clinical samples such as *E. coli*, *S. aureus*, and *Enterococcus faecalis*. The third type includes resistant strains including extended-spectrum beta-lactamase (ESBL) positive *Escherichia coli*, Methicillin-resistant *S. aureus* (MRSA), vancomycin-resistant *E. faecalis* (VRE), Methicillin-resistant S. epidermidis (MRSE), ampicillin-resistant *E. coli* O157:H7, erythromycin-resistant S. pyogenes, Teicoplanin resistant S. Pneumoniae, and levofloxacin-resistant *S. aureus*. Some of the studies determined antibacterial, bactericidal, and bacteriostatic effects of the NPs according to Clinical Laboratory Standards Institute (CLSI) methods such as Kirby-baur disk diffusion or well diffusion methods. Also, The minimum bactericidal activity (MBC) and minimum inhibitory concentrations (MIC) were performed by broth micro/macro dilution assay. Different studies presented an innovative research by combination of two or three NPs to evaluate antibacterial synergistic effects such as Au–Ag, Cu/Ni bimetallic, Cu/Zn in carbon nanofibers, Fe₃O₄@Au, and Au/Pt bimetallic. Another creative idea to increase the effectiveness of antibiotics is combining antibiotics with nanomaterial compounds, which are called nano-antibiotics. For example, when vancomycin conjugated with silver NPs, its antimicrobial activity multiplies against multi-drug resistant organisms such as VRE and MRSE. One of the studies presented a new approach to bacteriologically synthesized bimetallic gold–silver NPs using γ proteobacterium, Shewanella oneidensis MR-1 that appears to be a promising nano antibiotic for overcoming bacterial resistance in the established bacterial biofilms. Another achievement is the use of natural agents or standard microorganisms to synthesize different NPs and evaluated their antibacterial activity against MDR bacteria and bacterial biofilms. For instance, the potential of using plant S. anacardium, Glochidion lanceolarium, and Bridelia retusa derived AgNPs, using panchagavya to synthesize gold NPs, synthesis of titanium dioxide NPs using Azadirachta indica leaf extract, or using the aqueous extract of Phoenix roebelenii palm leaves to synthesize zinc oxide NPs. Some of the studies focused on the physical and morphological properties of the NP and presented that NP toxicities are depends on their chemical composition, size, crystalline phase, purity surface charge, shape, and bacterial strain. Besides, different researchers explain nano-sensitizer that enhanced their antibacterial activities through oxidative stress induced by ROS when they are exposed to different light sources such as near-infrared (NIR) or Ultraviolet (UV) irradiation. Moreover, various studies concentrated on the interaction between NPs and different parts of bacterial structures by attenuated total reflectance (ATR) Fourier transform infrared (FTIR) spectroscopy, and scanning/transmission electron microscope. More information was characterized in Table 1 and Fig. 2.

## 4 Discussion

Although MDR bacteria have increasingly common and pose a severe risk to world health, treatment options for the MDR bacteria with available antibiotics are insufficient. Particularly, the rise of pan-drug-resistant and extensive-drug-resistant bacteria worsens the current situation. Today, nanotechnology science is an alternative approach to control antibiotic resistance and chronic bacterial infections. Different studies investigated the potential antibacterial and anti-biofilm activity of the various nanomaterial.

Although the antibacterial mechanism of all NPs have not precisely identified, some studies described these mechanisms. For instance, Ag NPs have a bactericidal action based on inhibition of cell wall synthesis, protein synthesis mediated by the 30s ribosomal subunit, and nucleic acid synthesis [[30], [31], [32], [33], [34], [35]]. Also, Ultra small Au nano clusters could induce a metabolic imbalance in bacterial cells after the internalization, and leading to an increase of intracellular reactive oxygen species (ROS) production that kills bacteria consequently [36, 37]. Different studies documented light-enhanced antibacterial activity of TiO2 (≈1-Log reduction at 5 mg L-1) through oxidative stress that induced by ROS and impairment of bacterial cell membrane integrity. TiO2 NPs generated three types of ROS (superoxide radical, hydroxyl radical, and singlet oxygen). In addition, antibacterial activity of some antibiotics such as beta lactams, cephalosporin, aminoglycosides, glycolpeptides, erythromycin, clindamycin and tetracycline can increased by sub-inhibitory concentration of TiO2 NPs [[38], [39], [40], [41]]. Other researchers described antibacterial function of Al2O3 NPs. This nanoparticle cause cell wall leaking, destroy cell membrane through structural changes in phospholipids, and interact with the cellular macromolecules. ATR-FTIR studies suggested the interaction of lipopolysaccharide and l-α-Phosphatidyl-ethanolamine with Al2O3 NPs [42,43]. Moreover, antibacterial activity ZnO NPs dependent on the size of the NPs, production of ROS, bacterial enzyme inhibition (β-galactosidase), accumulation in the cytoplasm or on the outer membranes, binding to the intracellular material, and disruption of bacterial cell wall [[44], [45], [46], [47], [48]]. Different studies introduced Cu NPs as a putative anti-biofilm agent by decreasing biofilm hydrophobicity and exopolysaccharides structures. Cu NPs induced the antibacterial behavior by producing ROS and photothermal conversion in the hydrogel form under laser irradiation [[49], [50], [51]]. Several studies reported the importance and mechanism of action of copper nanoparticle in inhibition of bacterial infection. For example, CuO NPs killed bacterial cells by membrane lipid per-oxidation and chromosomal DNA degradation. Therefore, copper sulfide nanoclusters inhibit bacterial species at 5.5 μg/ml under NIR laser irradiation (980 nm, 1.5 W cm–^2^, 5 min) by heat ROS generation. In Cu/Ni bimetallic NPs, Cu NPs show a bactericidal effect while Ni NPs and bimetallic Cu–Ni NPs exhibit bacteriostatic effects [[52], [53], [54]]. Varies studies discussed antibacterial efficacy of Se NPs. For instance, SiO2-Cy-Van nanoprobes system exhibit outstanding photothermal antimicrobial activity that induces significant disruptions of the bacterial cell wall and membrane of VRE. Similarly, disrupting the bacterial cell membrane, induced a high level of ROS in the bacterial cell, and caused the oxidative damage to macromolecules [[55], [56], [57]]. According to recent studies, Fe_3_O_4_ NPs showed antibacterial activity concluded membrane dysfunction by magnetic core (Fe3O4)-shell (SiO2–NH2) NPs (MCSNPs), or damage to biological macromolecule by oxidative stress [[58], [59], [60]]. Besides Fe₃O₄@Au NPs composite damage to the bacterial cell wall by increased plasma membrane permeability, disruption of bacterial metabolism, interacts with bacterial proteins, and interfere with electron transport during oxidation of nicotinamide adenine dinucleotide by generation of ROS in bacteria [61]. Other NPs with strong antimicrobial effects include Au/Ag. On one hand, the main mechanism of Au–Ag NPs is the inactivation of proteins and enzymes necessary for the production of ATP, on the other hand different studies indicated Au/Pt bimetallic NP damage bacterial inner membrane and the increase of intracellular ATP levels, but do not involve the generation of ROS. A surprise here is that the antibiotic mechanism does not involve ROS at all/Au Pt NPs showed selective toxicity to bacteria but not to mammalian cells. Besides, studies showed that assembly of Au–Ag NPs onto the bacteria surface can released Ag+, generating ROS, and destroying the bacterial cells. One of the most interesting studies using Au/Ag bimetallic NPs for designing silicon 2, 3-naphthalocyanine dihydroxide (Nc) and Van functionalized silica-encapsulated, silver-coated gold NPs (Au@AgNP@SiO2@Nc-Van) system to enhanced efficacy of the Vancomycin and increase photo inactivation of the VRE. This novel nanosystem integrating surface-enhanced Raman scattering (SERS) imaging and noninvasive antimicrobial photodynamic therapy has huge potential for applications in theranostics with regard to VRE management [49,[62], [63], [64]]. Other research conducted on graphene oxides NPs have shown that reduced graphene oxide (rGO)-iron oxide NPs (rGO-IONP) under near-infrared laser created large amounts of ROS and inhibited MRSA. Moreover, the sub-inhibitory concentrations of this nanoparticle can inhibit bacterial biofilm formation in both gram-negative and gram-positive bacteria, especially in form of GO-Ag bimetallic [[65], [66], [67]]. As well as Ni-NPs Effecting on permeability and proper transport of the plasma membrane, penetrate inside the bacteria, and believed to damage them by interacting with phosphorous and sulfur containing compounds such as DNA [68,69]. The use of nanomaterials, and more specifically metal and metal oxide NPs, have recently received much interest as a potential method for detecting and treating MDR bacterial infections [70,71]. Inherent benefits of metal-based NPs include their very high surface area, simplicity of surface modification, perfectly adjustable size, and special mechanical, optical, chemical, and electromagnetic properties. Metal-based NPs' antibacterial activity studied intensively during the last two decades [[72], [73], [74]]. Although silver and copper have been used for thousands of years as antibacterial agents to preserve food and purify water [75,76]. Metal-based NPs have an antibacterial effect primarily via releasing metal ions, direct disruption of bacterial membranes, producing ROS, or a combination of the abovementioned processes. NPs made of noble metals, such as Au, Ag, and Cu, have also been reported to exhibit powerful antibacterial properties. It has been shown that some metal NPs may inhibit the growth of bacteria by releasing metal ions that bind to nitrogen, oxygen, or sulfur in biomolecules [76,77]. Ag NPs, for instance, show potent antibacterial action by releasing Ag^2+^ ions. Moreover, ROS produced by metal-based NPs like those that Au, ZnO, and Cu may lead to oxidative stress and have a lethal impact on bacterial genetic material or cellular membranes. Furthermore, Metal-Based NPs with distinct optical characteristics based on size and morphology have higher antibacterial activities through light irradiation. Photodynamic Effects of Metal-Based NPs is the name of this procedure. These NPs may also transform light into heat and boost photothermal impact to denature bacterial proteins [[78], [79], [80]]. Another health crisis is the emergence of biofilms. The biofilm was first identified as the primary type of bacterial presence in many diverse settings in the late 1970s [81]. One of the biggest problems in the medical profession is managing biofilm infections. Biofilms are complex, matrix-enclosed, and differentiated colonies of microorganisms adhering to biological or inert surfaces [82]. The biofilm can react cooperatively to potential dangers and changes in the environment. Accordingly, biofilm maturations permit bacteria to live and flourish in environments and medical devices that would otherwise be harmful. The most challenging obstacle in treating biofilm-associated infections is defeating antibiotic tolerance and resistance [83]. Multiple mechanisms of antibacterial tolerance and resistance have been proposed, including restricted diffusion of antibacterial drugs in the biofilm matrix, antibacterial drug deactivation in the biofilm's outer layers via enzymatic modification or binding to matrix parts, and the presence of niches in the biofilm matrix with insensitive cells, such as persisted and starved cells [[84], [85], [86], [87], [88]]. Innovative approaches that can defeat these resistance mechanisms are urgently needed. The utilization of NPs for the delivery of antimicrobial drugs is one strategy that is attracting a lot of attention [89,90]. Over the last ten years, there has been a steady increase in the number of studies concerning the use of nanomedicines in biofilm eradication and the prevention of their development [[91], [92], [93], [94]]. Regarding the prevention of biofilm infection, it is crucial to take into account the polymers' size (which varies between 5 and 100–200 nm), shape, surface (within a narrow window of positive charge densities), and interior characteristics of the resultant NPs. Metal oxide NPs, for instance, have potent antimicrobial effects and may prevent the growth of biofilms. Additionally, heavy metal NPs like Ag with diameters smaller than 20 nm may kill significant gram negative/positive planktonic bacteria and synergistically affect antibiotics [[95], [96], [97], [98]]. As well, carbon-based nanomaterials with various potential preparations have thermal, electronic, and bacterial killing capabilities. Moreover, mesoporous silica NPs are ideal for transporting antimicrobials. Mesoporous silica NPs have a unique porous structure, stable and rigid framework, and tunable particle size. These features have shown adequate effects on biofilms [83]. On the other hand, liposomes play a unique function in destroying biofilms by fusing with bacterial membranes, albeit the exact processes by which they do this are still unclear. Several studies have demonstrated that modified antibiotics loaded with phosphatidylcholine liposomes are more strongly attracted to negatively charged bacterial cell surfaces, better penetrate biofilms, and kill bacteria [83,99]. Although the design and introduction of NPs with antibacterial effects have made good progress, there are also limitations in this field. Many of these NPs have only been studied *in-vitro* and considering them as an antibacterial and anti-biofilm agent requires the expansion of research *in-vivo*. For example, if a nanoparticle in the culture medium shows a potential antibacterial effect against *P. aeruginosa*, it should also show these effects on infected wounds in animal models. In addition, if a nano particle introduced as an anti-biofilm agent, it is better to investigate its performance in medical devices such as venous catheters, where microbial biofilm formation occurs. In addition, NPs that have been effective in vivo conditions, it is better to enter the clinical trial phases through careful consumption planning. By reviewing and comparing the research conducted in the field of NPs with antibacterial properties, it is clear that many of these studies have only used standard microbial strains. While these studies should be done on clinical samples isolated from patients, especially antibiotic-resistant agents such as carbapenem-resistant *Acinetobacter*, ESBL producing and carbapenemase-resistant Enterobacteriaceae, in addition to standard strains. On the one hand, studies that investigate the antibacterial effect of NPs state that they have been implemented based on the international standards of Clinical and Laboratory Standards Institute (CLSI) or European Committee on Antimicrobial Susceptibility Testing (EUCAST). While in these standards, the results of sensitivity or resistances of only a number of microbial strains to certain antibiotics, not NPs, have been proposed. The best way to refer to these standards is to check the simultaneous performance of NPs against a microbial strain in the presence of antibiotics suggested by the standards as a control. Based on research in the field of NPs with antibacterial properties, it is conceivable that studies have focused only on a specific group of infectious agents (Table 1). While today, the challenging issue is fastidious, anaerobic bacteria, and antibiotic resistances related to them. These include oral and dental infections and biofilms caused by drug-resistant bacteria, MDR and extensively drug-resistant (XDR) strains of *Mycobacterium tuberculosis*, or pan-drug-resistant *A. baumannii* and these types of resistances must be considering in the future research. In terms of the method, some studies have reported the antibacterial effects based only on observing the lack of growth in the well diffusion or disc diffusion method. Although the advances made by nanotechnology in the field of controlling microbial agents, so far, they have shown poor performance in conditions such as bacteremia, septicemia, and bacterial meningitis, and future studies should consider these problems. Nevertheless, one of the disadvantages of using some NPs is the possibility of their toxicity to human cells, which may enter the circulatory system, lungs, liver, spleen, and brain, and their toxic effects would investigate in future studies. However, scientific advances in the nano field regarding the identification of NPs with antibacterial and anti-biofilm effects can not be ignored. Finally, to overcome the microbial biofilm and defeat antibiotic resistance, future studies should pay attention to these mentioned limitations.

## 5 Conclusion and outlook

MDR pathogens are an emerging public health concern that makes it difficult to treat many illnesses associated with healthcare using current and available antibiotics. Undoubtedly, nanotechnology is a revolution in the field of medicine and drug delivery, especially in drug-resistant infectious diseases; it plays an important role in restoring the activity of antibiotics against resistant bacteria. Nanomaterial offer a potential strategy for controlling illnesses brought on by these infections. Antibacterial NPs may target a wide range of biomolecules, potentially slowing or stopping the spread of MDR. A complete understanding of the pharmacodynamics of NPs, bio distribution pharmacokinetics, in-vivo, and in-silico effects, pharmacodynamics of NPs, and unique physicochemical characteristics are also required for the conversion of NPs to medical applications. Specific combinations of NPs and antibiotics may help to prevent resistance or push resistant bacteria back to drug sensitivity. Administration of antimicrobial agents based on NPs can improve the level of treatment indicators. Additionally, clinical data on nanomaterial-based antibacterial properties are limited; therefore, further in-depth investigations including in-vivo results, are required to successfully preparation of nanomaterials for medicinal applications in combating MDR pathogens. It is vitally necessary to develop novel techniques for treating device-associated biofilms since the recurrence of bacterial infection during revision operations continues to be clinically significant. While several nanomaterials have shown promise in limiting biofilms' development, not all of them can eliminate biofilms. This is primarily because bacterial biofilms include a highly resistant bacterial colony encased in a matrix of their own secreted extracellular polymer material (EPS). Treatment of these biofilms requires eliminating these bacteria as well as their EPS, making it far more complex than preventing biofilm formation. Specific nanomaterials have been demonstrated to have inherent biofilm-eliminating abilities; however, it is anticipated that the treatment effectiveness would enhance greatly when intrinsic bactericidal or EPS-lysing abilities are combined with stimulation such as local pH, magnetic fields, or light. Based on studies, so far, various NPs with a strong ability to control bacterial infections by overcoming biofilm and antibiotic-resistant strains have been discovered, which can transform the future of prevention and treatment. It seems that development of a drug delivery system based on nanostructures will be an attractive and effective way to overcome antibiotic resistance and biofilm if the toxic effects of NPs investigated and their safety guaranteed.

## Data availability statement

Data included in article/supp. Material/referenced in article.

No additional information is available for this paper. Declaration of competing interest.

The authors declare that they have no conflict of interest.

## Ethics approval and funding

Not applicable.

## Additional information

No additional information is available for this paper.

## Author contribution statement

Conceived and designed the experiments: Farhad Moradi, Aida Bazrgar.

Performed the experiments: Farhad Moradi, Aida Bazrgar, Arshin Ghaedi.

Analyzed and interpreted the data: Zahra Fooladfar, Arshin Ghaedi.

Contributed reagents, materials, analysis tools or data: Farhad Moradi, Zahra Fooladfar.

Wrote the paper: Farhad Moradi, Aida Bazrgar, Arshin Ghaedi, Zahra Fooladfar.

## Declaration of competing interest

The authors declare that they have no known competing financial interests or personal relationships that could have appeared to influence the work reported in this paper.
